# Dyspepsia of Liquids

**Published:** 1873-01

**Authors:** John C. Thorowgood


### DYSPEPSIA OF LIQUIDS.

BY DR. JOHN C. THOROWGOOD.

The cases here briefly recorded have seemed to me good illustrations of that indigestion of liquids which has been carefully described by M. Chomel, in his work on dyspepsia.

CASE I.—James McC., aged thirty, a pale, dark, intelligent man, came under my care September 14th, 1861, complaining generally of dyspeptic symptoms, and specially of the great uneasiness caused by the presence of any amount of liquid in his stomach. Liquids in the slightest degree acid were most distressing to him, and at intervals he had attacks of sour pyrosis. He complained much of dryness of mouth, with dry skin and costive bowels; urine loaded with lithates, but free from traces alike of sugar or albumen. No loss of flesh; pulse slow and soft; nothing irregular to be found in heart or lungs; no sort of tumor or thickening about pylorus of stomach, but on greatly vibrating this organ, fluid was heard splashing about within it, and this sound could be always produced irrespective of any liquid recently drunk. The stomach was much distended. The early treatment of this case consisted in the use of alkalies, with bismuth and various bitters, but no improvement resulted; the only noteworthy feature was the effect of a pill of extract of opium at night, which regulated the action of the bowels so completely that the patient asked for the pills as *aperient* pills.

Often at his visits this man drew my attention to the quantity of liquid always present in his stomach, and at last we agreed that he should drink as little as possible, and take no other medicine than a powder of rhubarb and magnesia every morning. From this time he steadily improved, and after about two months' treatment he was let go cured, and six months afterwards was still in good health.

CASE II.—A man aged fifty-three years, dark, sallow and very hypochondriacal, had been dyspeptic for eighteen months, and had taken quantities of physic. He often has pyrosis, has large sodden tongue, costive bowels, and dry skin; urine dark in color, specific gravity 1030, acid, and nothing abnormal chemically or microscopically. The indigestion of liquids was not so marked in this case as in the one preceding, but no medicine gave relief till the dry plan of diet was enforced; then he amended considerably, and I lost sight of him.

CASE III.—A young, healthy-looking man, aged twenty years, by trade a bricklayer, came to me complaining of faintness and palpitation of the heart, and said he had got water in his chest, for he could feel it. Examination of the chest showed the lungs and heart to be in every respect quite healthy; the stomach is large and dilated, and when he shakes, the splashing noise of liquid therein is plain enough. He says he can produce this noise at any time. This patient has a dry skin; large sodden tongue; pulse 80; urine clear. No waterbrash.

So far as treatment has gone during one month with alkalies, bismuth and various tonics, little good has been done. The dry-diet plan is the only means of effecting a regular cure; but as this involves a pretty complete abstinence from beer, the patient can hardly be made to give it a fair trial. That the less he drinks the better he is, however, is a fact already well proved.

At first sight, this sort of cases may appear of a pretty ordinary kind; but what I claim as their characteristic features are these: They are cases of obstinate dyspepsia, marked specially by the discomfort of feeling the stomach always distended as with fluid, while the mouth is usually dry, thirst troublesome, and yet drinking always aggravates the discomfort. There is no acute pain, and the patient rarely gets thin. Movement of the patient produces that splashing noise, or "*clapotement*," as Chomel calls it, significant of the presence of wind and water in the stomach; and this can be produced at any time by the patient. Lastly, there is the almost certain amelioration of the symptoms under the influence of the dry plan of diet; and till that is followed, no medicine does any good whatever.

It is necessary to seek carefully for any organic disease, for if there be stricture or thickening about the pylorus, it will not add much to the doctor's credit to promise a certain cure if the dry plan is followed; relief there may be, but not cure. Chomel says of one of his cases: "J'ai vu un autre individu, atteint de dyspepsie des liquides, qui, en appuyant avec quelque force la main sur l'epigastre, faisait remonter dans sa bouche plusieurs onces d'un liquide aquex; mais ce fait a ete unique pour moi." I have sought to elicit this symptom in the cases recorded, and in others, but have not been able to produce it.

At times, fits of fainting, with irregular action of the heart, are prominent symptoms in these cases of dyspepsia of liquids, the cause being due to the state of the stomach. Marked relief is obtained to these symptoms on the dry-diet plan, and this confirms the soundness of the practice of not allowing patients with heart disease to live much on liquids and slops, but rather to feed them on easily digested solids, such as mutton, chicken, boiled fish and game. These are all forms of solid food suitable in cases of dyspepsia of liquids. Milk does not seem otherwise than beneficial, but much of farinaceous or vegetable food should be avoided.

The patient must bear a certain amount of thirst as well as he can, and when he does drink, must take but very small quantities at a time, and not drink for an hour or more after he has taken his meal of solid food. Weak whisky-and-water, sherry wine, and toast-and-water, are among the least objectionable drinks; and sometimes a small cup of pure beef-tea, free from any farinaceous admixture, will suit well. One of my patients found a wineglass of good stout to agree well, and relieve his thirst.

Compared with diet, medicine is but a secondary matter; but a dose of Gregory's powder in the morning, and a tonic mixture of rhubarb and tincture of nux vomica twice a day, I have found serviceable. Mild saline purgation I have tried, but its effects are very uncertain, relieving at times slightly, at other times adding decidedly to the discomfort, and it is not a method of treatment to be recommended. A pill of opium at night, when there is much nervousness and hypochondriasis, is certainly beneficial, and a pill of bitter extract, or of galbanum and assafœtida, twice a day, may be used with advantage.

It is rare for the patients to emaciate, provided the case is uncomplicated. In one of Chomel's cases, however, he noticed a degree of emaciation that caused great anxiety, being an unusual symptom, and likely to indicate organic visceral disease. The dry regimen was enforced, and the lady got quite well, though any over-indulgence, even in such a fluid as plain water, would be followed by a return, in some degree, of the unpleasant symptoms.—*Lancet.*

